# Potato virus X-mediated constitutive expression of *Plutella xylostella PxSDF2L1* gene in *Nicotiana benthamiana* confers resistance to *Phytophthora parasitica* var. *nicotianae*

**DOI:** 10.1186/s12870-021-02854-5

**Published:** 2021-02-05

**Authors:** Ivis Moran-Bertot, Lianet Rodríguez-Cabrera, Orlando Borras-Hidalgo, Siliang Huang, Yunchao Kan, Denis J. Wright, Camilo Ayra-Pardo

**Affiliations:** 1 Plant Division, Centre for Genetic Engineering and Biotechnology (CIGB), 10600 Havana, Cuba; 2 Shandong Provincial Key Laboratory of Microbial Engineering, School of Biotechnology, Qi Lu University of Technology, Jinan, 250353 Shandong People’s Republic of China; 3 China-UK-NYNU-RRES Joint Laboratory of Insect Biology, Nanyang Normal University (NYNU), Nanyang, 473061 Henan People’s Republic of China; 4 Department of Life Sciences, Imperial College London, Silwood Park campus, Ascot, Berkshire, SL5 7PY UK

**Keywords:** Crop protection, Disease resistance, Oomycetes, Agroinfiltration, ER chaperone

## Abstract

**Background:**

The *Plutella xylostella PxSDF2L1* gene was previously reported to enhance insect resistance to pathogen at high basal transcription rate. PxSDF2L1 shows similitude with the stromal cell-derived factor 2 (SDF2), an ER stress-induced chaperon protein that is highly conserved throughout animals and plants. The precise biological function of SDF2 is not clear, but its expression is required for innate immunity in plants. Here, we investigate whether a continuous expression of *PxSDF2L1* in *Nicotiana benthamiana* can similarly confer resistance to plant pathogen, particularly, the black shank *Phytophthora parasitica* var. *nicotianae*.

**Results:**

The *N. benthamiana* plants were inoculated with agrobacteria transformed with a PVX-based binary vector carrying the *PxSDF2L1* gene; similar agroinoculation experiments with a PVX vector carrying the *GFP* gene were used for controls. In pot trials, agroinfected *N. benthamiana* plants constitutively expressing *PxSDF2L1* showed a significant reduction of stem disease symptoms caused by the inoculation with *P. parasitica*, compared with controls.

**Conclusions:**

We confirm a role of *PxSDF2L1* in resistance to black shank, with a potential application to engineering active resistance against this oomycete in the commercial *N. tabacum* species and propose its evaluation in other crop families and plant pathogens.

**Supplementary Information:**

The online version contains supplementary material available at 10.1186/s12870-021-02854-5.

## Background

The stromal cell-derived factor 2 (SDF2) is an endoplasmic reticulum (ER)-resident protein that is highly conserved in plants and animals [1, 2]. SDF2-type proteins adopt a typical β-trefoil fold made up of three MIR motifs, which are also found in protein O-mannosyltransferase, inositol 1,4,5-trisphosphate receptor and ryanodine receptor [3]. In the sequence of SDF2-type proteins, three pairs of specific hydrophobic residues located at the triangular cap and the bottom and the middle layers of the barrel, respectively, maintain the β-trefoil structure [2].

The precise molecular function of SDF2 is not known. SDF2 is part, with ER-resident chaperones Hsp40 protein ERdj3, Hsp70 luminal binding protein (BiP) and other folding enzymes of the ER quality control (ER-QC) machinery, controlling the folding status of secreted and transmembrane proteins to ensure the delivery of functional molecules to their final destination [4, 5]. BiP is the central player of the multiprotein complex BiP/ERdj3/SDF2, while ERdj3 co-chaperone provides the BiP chaperone with unfolded polypeptides and regulates its activity [6]. SDF2 forms a stable complex with ERdj3 to assist BiP by directly inhibiting the aggregation of non-native proteins [7]. In human pancreatic cells, SDF2-like 1 (SDF2L1) protein retards the degradation of unfolded proteins by the ER-associated protein degradation (ERAD) machinery [8].

Plants respond to pathogens by mobilising many proteins among transmembrane immune receptors and secreted defensive proteins that rapidly overload the ER folding capacity and induce the ER stress which triggers a complex protective pathway, termed the unfolded protein response (UPR) [9]. One of the UPR targets is SDF2, whose expression is significantly induced in *Arabidopsis*, mice and humans [1, 2]. The UPR tries to restore ER homeostasis by reducing the number of proteins loaded into the ER and enhancing the ER-QC capacity (i.e. increasing the synthesis of ER chaperones and associated proteins) and ERAD activity [10–13]. UPR can also activate programmed cell death (PCD) when ER stress is excessive and prolonged [14]. Several ER-QC components, including SDF2, have been shown to have a significant effect on host immunity. For instance, the biogenesis and maturation of the glycosylated immune receptor EFR -a pattern recognition receptor (PRR) that recognises the bacterial elongation factor EF-Tu to confer pattern-triggered immunity (PTI)- requires the ER-QC complex BiP/ERdj3/SDF2 [15]. In *Arabidopsis*, T-DNA insertion mutants *sdf2–2* (SALK_141321) and *sdf2–5* (WiscDsLox293–296invI23) that express no SDF2 protein retain and degrade EFR in the ER. In rice, SDF2 has been recovered among ER-QC proteins interacting with XA21, a host PRR that confers PTI against *Xanthomonas oryzae* pv. *oryzae* (Xoo) [16, 17]. Knockdown of *SDF2* gene expression affected XA21-mediated resistance to Xoo, which indicated SDF2 participation is critical for XA21 function [17].

Black shank *Phytophthora parasitica* var. *nicotianae* is a root oomycete pathogen of economically important crops and forest trees worldwide for which no control method is yet available [18]. In plants from the *Nicotianae* family, including the model plants *Nicotiana benthamiana* and *N. tabacum*, *P. parasitica* affects mainly the roots and basal stem area, but all parts of the plant can become infected [19]. The most common symptoms of the disease are associated with the black base or shank of the stalk. The mechanisms used by *P. parasitica* to achieve host compatibility and promote infection are not fully understood. An effector protein PpRxLR2 has been found to suppress *N. benthamiana* immunity by targeting a yet unknown plant susceptibility gene [20]. While Breeze et al. [21] have shown ER to be an essential target of RxLR-type effectors, which induce a rapid reconfiguration of the organelle coincidently with the suppression of host defences and initial pathogen multiplication.

Previously, we showed increased basal mRNA levels of *PxSDF2L1*, the gene encoding for an SDF2-type protein homologue in the diamondback moth, *Plutella xylostella* (Lepidoptera: Plutellidae), were required for resistance to *Bacillus thuringiensis* (Bt) [22]. Given the highly conserved nature of SDF2-type proteins throughout the plant and animal kingdoms [1, 2] and its importance for the accumulation and function of immune receptors in the plasma membrane, we hypothesise the constitutive expression of *PxSDF2L1* could have a priming effect on host immunity, preventing pathogen growth at the early stages of infection.

The aim of the present study was to test whether the constitutive expression of *PxSDF2L1* in *N. benthamiana* can confer resistance to *P. parasitica*. We used a Potato virus X (PVX)-mediated expression system for rapid in planta verification of our hypothesis. A PVX construct carrying the *PxSDF2L1* gene was introduced into *N. benthamiana* plants by agroinfiltration; a PVX vector carrying the green fluorescent protein (GFP) insert was used to agroinoculate controls. The transcription of *PxSDF2L1* gene in the roots of PVX.PxSDF2L1-agroinfected plants was investigated by RT-PCR. Disease resistance was determined through *Phytophthora* pot trials. We found the systemic constitutive expression of *PxSDF2L1* in *N. benthamiana* confers protection against *P. parasitica*, with a potential application to engineering active resistance against this oomycete in the commercial *N. tabacum* species and propose its evaluation in other crop families and plant pathogens.

## Results

### PxSDF2L1 and insect homologues are SDF2-type proteins

**Fig. 1:**
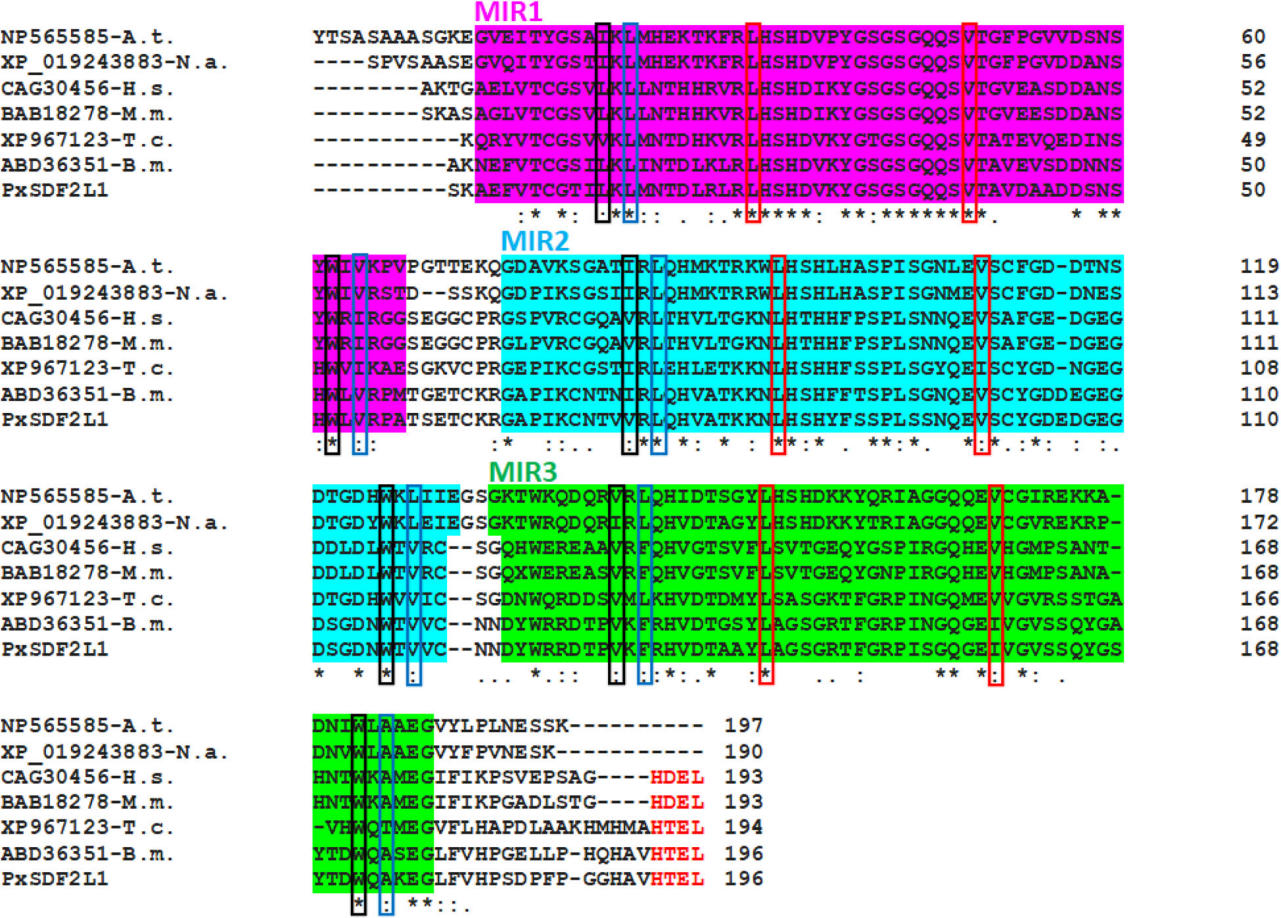
Multiple sequence alignments of plant, mammal and insect SDF2-type proteins. The sequences were aligned with Clustal Omega tool, using default settings (EMBL-EBI; https://www.ebi.ac.uk/Tools/msa/). For a better alignment, signal peptides predicted with ‘SignalP 4.1 Server’ (http://www.cbs.dtu.dk/services/SignalP/) [23] were removed from the sequences. The sequence of *Plutella xylostella* PxSDF2L1 has been published (GenBank accession no. HQ199329). The other SDF2 sequences are coded by their GenBank accession no. followed by the abbreviated name of the organism: A.t., *Arabidopsis thaliana*; N.a., *Nicotiana attenuata*, H.s., *Homo sapiens*; M.m.: *Mus musculus*; T.c., *Tribolium castaneum*; B.m., *Bombyx mori*. In the alignment, the stretch of amino acid residues encompassing each MIR motif has been highlighted in different colours: MIR1 in magenta, MIR2 in cyan, and MIR3 in green. Conserved residues at the interior of the β-trefoil barrel arranged in the bottom and middle layers are squared in black and blue, respectively. The key residues of the triangular cap are squared in red. The ER retention tetrapeptides ‘HDEL’ or ‘HTEL’ [24] appear in red

Multiple sequence alignments of PxSDF2L1 and insect homologues with plant and mammal SDF2-type proteins revealed the conservation of typical features of the SDF2 family in insects, such as the arrangement of MIR motifs in triplet and the critical hydrophobic residues essential for maintaining the β-trefoil structure (Fig. 1). In contrast to plants SDF2-type proteins, insect SDF2-like sequences contain an ER-retrieval signal at the carboxyl-terminal like mammalian SDF2L1 [1], which indicates ER as their only subcellular localisation.

### *PVX-mediated systemic expression of* PxSDF2L1 *in* N. benthamiana *plants*

**Fig. 2:**
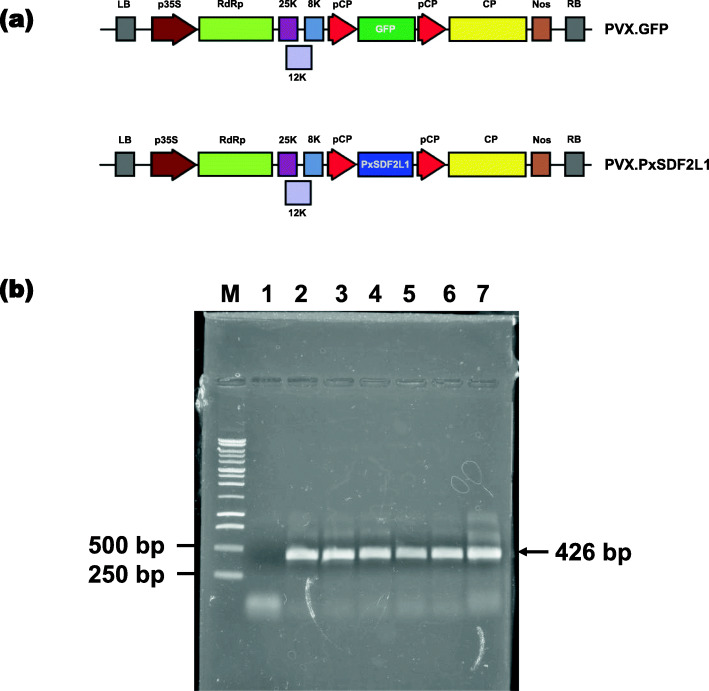
PVX-mediated recombinant gene expression in *N. benthamiana*. **a** Schematic representation of PVX.GFP (control) and PVX.SDF2L1 binary constructs carrying the recombinant genes *gfp* and *PxSDF2L1*, respectively. In the scheme: LB and RB, left and right T-DNA border sequences; p35S, 35S promoter of cauliflower mosaic virus; RdRp, PVX RNA-dependent RNA polymerase gene; 25 K, 8 K, and 12 K, PVX movement protein genes; pCP, subgenomic promoter sequence (duplicated); CP, PVX coat protein gene; Nos, nopaline synthase transcriptional terminator. **b** RT-PCR reaction of *PxSDF2L1* in RNA samples from the root of plants agroinfected with recombinant PVX vectors, 21 d.p.ai. Lane 1: PVX.GFP plant (negative control); Lanes 2–7: PVX.PxSDF2L1 plants. M, 1 kb DNA ladder

The expression of *PxSDF2L1* in *N. benthamiana* was investigated using a PVX-derived binary vector carrying the recombinant insert (PVX.PxSDF2L1); similar infections were carried out in parallel with a control PVX binary vector carrying the *GFP* gene (PVX.GFP) (Fig. 2a). First, the progression of PVX viral infections was monitored through the detection of reporter GFP in systemic uninoculated-upper leaves of PVX.GFP-challenged plants via confocal laser scanning microscopy (CLSM). As expected for the permissive host species *N. benthamiana* [25, 26], a strong systemic accumulation of PVX.GFP (based on the GFP-derived green fluorescence signal) was detected 21 days post-agroinfection (d.p.ai.) in all tested PVX.GFP plants (Fig. S1). Following CLSM, RT-PCR experiments investigated *PxSDF2L1* systemic expression in the root tissues of PVX.PxSDF2L1-agroinfected plants. A cDNA fragment of the expected size (~ 426 base pairs) was consistently amplified from roots of PVX.PxSDF2L1 plants and not from that in PVX.GFP plants (Fig. 2b). The amplified cDNA was verified as a fragment of *PxSDF2L1* by DNA sequence analysis (Data not shown). PVX vector DNA contamination was not detected after 40 cycles of PCR on RNA samples from PVX.PxSDF2L1 plants (Fig. S2).

### *PVX.PxSDF2L1-challenged* N. benthamiana *plants are resistant to P. parasitica infection*

**Fig. 3:**
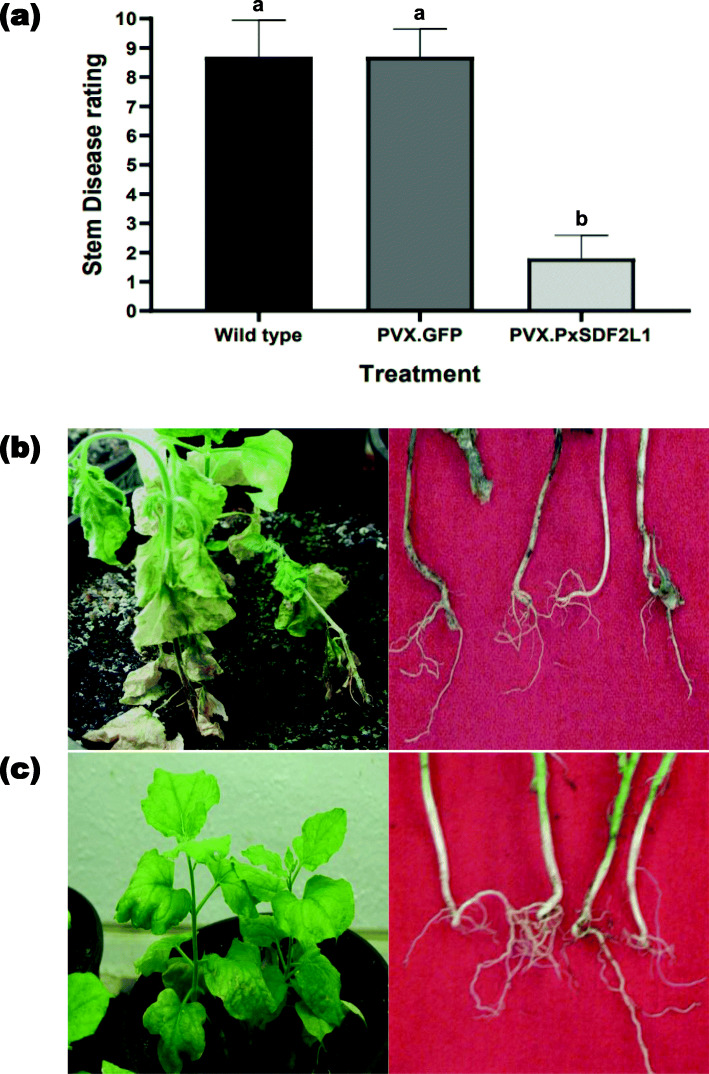
Glasshouse evaluation of PVX.PxSDF2L1-agroinfected *N. benthamiana* plants inoculated with *P. parasitica*. **a** Quantitative evaluation of *N. benthamiana* plants with stem disease rating represented using a 1–10 linear scale, where 1 was no disease and 10 was a dead plant [27] for wild type, PVX.GFP- or PVX.PxSDF2L1-agroinfected plants (21 d.p.ai.) inoculated with *P. parasitica* at seven d.p.i. Bars show the means ± SE of three independent experiments. In each experiment, 12 plants were used per treatment. The means were compared using a one-way ANOVA test with Tukey’s post-test at *P* < 0.05. Treatments not sharing a common letter were significantly different (*P* < 0.0001). Plant and stem phenotypes of (**b**) control (wild type and PVX.GFP-) and (**c**) PVX.PxSDF2L1-challenged plants at seven d.p.i. with *P. parasitica*

*Nicotiana benthamiana* is a host of *P. parasitica*. Wild type, PVX.GFP- and PVX.PxSDF2L1-agroinfected *N. benthamiana* plants, 21 d.p.ai., were inoculated with the isolate PpnIIT23 of *P. parasitica* race 0 and disease symptoms scored at 7 days post-inoculation (d.p.i.). Quantitative evaluation of *Phytophthora* pot trials revealed that stem disease symptoms occurring in PxSDF2L1 plants were significantly reduced compared with those in PVX.GFP and wild type plants (F_2,27_ = 154.1, *P* < 0.0001) (Fig. 3a). There was no significant difference in stem disease symptoms in PVX.GFP and wild type plants (F_9,9_ = 1.74, *P* = 0.42), indicating that nonspecific interactions with the PVX vector did not affect susceptibility to *P. parasitica*. While control (wild type and PVX.GFP) plants developed severe stem disease symptoms and damping-off associated with *P. parasitica* (Fig. 3b), only marginal symptoms were detected on PVX.PxSDF2L1 plants (Fig. 3c).

## Discussion

*Phytophthora parasitica* is considered one of the most devastating oomycete plant pathogens, causing severe damage to a broad range of host species, some of which are economically important crops [18, 19]. In the present study, we have constitutively expressed the *P. xylostella PxSDF2L1* gene in *N. benthamiana* using a PVX-based binary vector and demonstrated that it conferred resistance to *P. parasitica* during pot trials in glasshouses. In insects, SDF2-type proteins have been poorly investigated; however, as mentioned in the introduction, studies have suggested functional conservation of the SDF2 family in animals and plants. In our study, multiple sequence alignments of PxSDF2L1 and other insect homologues with SDF2 proteins in plants and mammals showed insect sequences following the very structural patterns of the SDF2 family. However, the presence of ER retrieval signals in mammals’ and insects’ SDF2 sequences but not in plants’ SDF2 sequences indicates ER retention of SDF2 in the latter depends on specific binding partner(s).

*Phytophthora parasitica* is a hemibiotroph pathogen, which means it starts infection as biotroph establishing close contact with living host cells to induce their death later and becomes necrotrophic [18, 19]. The *A. thaliana* ethylene-responsive factor 19 gene (*AtERF019*) mediates plant susceptibility to *P. parasitica* through suppression of PTI [28]. *P. parasitica* have developed strategies to suppress PTI through ER-localised effectors during the biotrophic relationship [21]. Transcriptomic analysis of *N. tabacum*’s molecular response to *P. parasitica* found defence to oomycetes and other essential plant defence mechanisms were suppressed during the interaction [29]. The constitutive expression of *PxSDF2L1* in *N. benthamiana* could provide plants with a defensive advantage in the early stages of *P. parasitica*’s biotrophic interaction, allowing a rapid accumulation and function of as yet unknown ER-QC client PRR(s) mediating the immune response against this root-infecting species. Previously, we showed increased basal expression of *PxSDF2L1* in Bt-resistant populations of *P. xylostella* was part of a pre-activated molecular defence mechanism of the insect to this pathogen [22]. More experiments are needed to identify PxSDF2L1’s interacting partner(s) in the ER involved in downstream pathogen resistance mechanisms.

In our study, an intriguing issue is whether the existence of an ER retrieval signal in PxSDF2L1 has contributed to *P. parasitica* resistance in PVX.PxSDF2L1-agroinfected *N. benthamiana* plants. While SDF2 has been identified as an ER-resident protein in *Arabidopsis* [2], how it is retained in this plant organelle and under what conditions remain poorly understood. The recognised binding partner of SDF2, ERdj3B also lacks an ER retrieval signal [15, 30], and in mammals, it can be secreted under ER stress conditions with unfolded conformers contributing to proteostasis at the extracellular space [31]. The binding of ERdj3 increases the stability of SDF2, which is otherwise a short-lived protein [7]. The SDF2-ERdj3B complex prevents unfolded proteins aggregation during the interaction with the UPR’s master regulator BiP, which might be involved in retaining both proteins into the plant ER. BiP is also the target of some *Phytophthora* ER-localised effectors, such as PsAvh262 from *P. sojae* [32]. The interaction BiP-effector has been suggested to decouple the SDF2/ERdj3B/BiP complex [32, 33], which might cause the release and secretion of SDF2 and ERdj3b, with negative consequences for the stable accumulation of PRRs at the plasma membrane and downstream defence responses. PxSDF2L1 chaperone has its ER retrieval signal and could rescue the system while BiP stays occupied with effectors. This explanation needs further investigation.

In summary, we have shown constitutive expression of *PxSDF2L1* gene in *N. benthamiana* confers protection against *P. parasitica* and propose its potential application to engineering active resistance against this oomycete in the commercial species *N. tabacum*. Future work with *PxSDF2L1* involves obtaining plants that stably express the gene for a more precise determination of its role in Black Shank resistance.

## Conclusions

The *P. xylostella* PxSDF2L1 shows similitude with ER stress-induced SDF2-type proteins from animals and plants, and the gene was previously found to enhance insect resistance to pathogen at high basal transcription rates. In the present study, we produced a systemic constitutive expression of *PxSDF2L1* in *N. benthamiana* plants with the aid of a PVX-based vector system. Further, *N. benthamiana* plants expressing the *PxSDF2L1* gene showed a significant reduction of stem disease symptoms caused by the inoculation with *P. parasitica* in pot trials, compared with controls. Our results suggest the potential application of *PxSDF2L1* to engineering active resistance against this oomycete in the commercial *N. tabacum* species. Future studies should assess the *PxSDF2L1* gene in other crop families and plant pathogens.

## Methods

### Plant material

The experimental research on plants was performed in strict accordance with the guidelines approved by the CIGB’s Plant division. The *N. benthamiana* seeds were obtained from the Tobacco Research Institute in Havana, Cuba. *Nicotiana benthamiana* plants were grown in a containment glasshouse (CIGB) at 25 °C until agroinfiltration experiments.

### Construction of PVX.PxSDF2L1

The *Escherichia coli* XL-1 blue strain [34] was used for all standard molecular biology techniques. The source for *P. xylostella PxSDF2L1* gene (GenBank accession no. HQ199329) was a plasmid carrying the full-length *PxSDF2L1* cDNA sequence (1111 bp), which had been obtained by rapid amplification of cDNA ends (RACE) [22]. A 676-base pair (bp) PCR fragment was amplified from *PxSDF2L1* cDNA template using mutagenic oligonucleotides (forward) 5′-atcgatgttacaggaatattacagtatg-3′ and (reverse) 5′-gtcgacttataactcagtatgaactgc-3′, where underlined sequences correspond to added restriction sites for ClaI and SalI endonucleases, respectively. The amplified product comprising the open reading frame of the gene was ClaI / SalI digested, purified, and ligated into the ClaI / SalI restriction sites of the PVX-based binary vector pGR106 (GenBank accession no. AY297843) [35, 36] obtained from Sir David Baulcombe (Plant Department, University of Cambridge, UK). Resulting recombinant PVX.PxSDF2L1 clones were identified by colony PCR and confirmed by DNA sequence analysis (Macrogen).

The PVX.GFP plasmid carrying the GFP insert cloned into the AscI and NotI restrictions sites of pGR106 [26] was obtained from Dr. Eleanor M Gilroy (James Hutton Institute, Dundee, UK) and used as an indicator of the progression of viral infection and potential virus-induced changes in *N. benthamiana* plants.

### *Agroinfiltration of* N. benthamiana *with PVX-derived binary vectors*

*Agrobacterium tumefaciens* strain GV3101 [37] harbouring helper plasmid pSoup (pJICSa_Rep) was transformed with PVX.PxSDF2L1 or PVX.GFP vectors by direct transformation [38] and grown in Luria Bertani (LB) medium supplemented with kanamycin at 50 μg/ml. *Agrobacterium* infection of *N. benthamiana* with PVX.PxSDF2L1 or PVX.GFP was performed as described by Voinnet et al. [39]. Briefly, overnight agrobacterial cultures (20 ml) of each construct were centrifuged at 2250×g for 10 min at 16 °C and cell pellets resuspended into 5 ml of a freshly-made and filtered Agromix solution [10 mM 2-(N-morpholino) ethanesulfonic acid (MES), pH 5.6; 10 mM MgCl2; 0.150 mM acetosyringone]. The agrobacterial suspensions were left static at room temperature in the dark for 2 h. Inocula (OD600 ~ 0.2) prepared by diluting agrobacterial suspensions in Agromix were then gently infiltrated into the lower leaf surface of six-leaf stage *N. benthamiana* plants using a 1-ml syringe (without a hypodermic attached). Agroinfiltrated plants were placed under controlled growth conditions of 16:8 h (light: dark) cycle, 50–60% humidity and 16–21 °C temperature.

### Detection of GFP

Systemic uninoculated leaves of PVX.GFP-infected plants were analysed for green fluorescence 21 d.p.ai. with a Zeiss LSM 700 confocal laser scanning microscope. GFP’s excitation and detection windows were set as 488 nm and 500–600 nm, respectively. Systemic uninoculated leaves from PVX.PxSDF2L1-infected plants were used as the negative control.

### Total RNA extraction and RT-PCR experiments

Roots from plants challenged with PVX.PxSDF2L1 or PVX.GFP (six plants each) were collected 21 d.p.ai. followed by washing with distilled water to remove soil from their surface. The collected roots were cut into small pieces and immediately frozen in liquid nitrogen (− 196 °C) followed by grinding in precooled mortars and pestles. Total RNA was isolated with the SV total RNA isolation system (Promega); purified RNA samples were digested with the RNase-free DNase I enzyme included in the kit for 30 min at 37 °C for removing contaminant PVX vector DNA followed by ethanol precipitation in the presence of 0.3 M NaOAc pH 5.2. PCR on the RNA samples with specific primers (5′-gaaacctcctcggattccat-3′; 5′-tctccaaatgaaatgaacttcc-3′) for a 312-bp fragment in the 35S promoter of cauliflower mosaic virus (p35S) in PVX-based binary vectors (PVX.PxSDF2L1 or PVX.GFP) was used to confirm the efficiency of DNase I digestion. RNA integrity was verified by 1% agarose gel electrophoresis stained with GelRed (Biotium), and RNA concentration was measured using a NanoDrop 2000 Spectrophotometer (Thermo Scientific).

Primers (forward) 5′-aaggcagaattcgtgacgtg-3′ and (reverse) 5′-tgccaaatatgctgcagtgt-3′ that together amplify a 426-bp *PxSDF2L1* cDNA fragment were used in RT-PCR on DNA-free RNA templates (1 μg) of PVX.PxSDF2L1-infected plants with the AccessQuick RT-PCR system (Promega). DNA-free RNA samples (1 μg) from PVX.GFP-infected plants were used as the negative control.

### Phytophthora *pot trials*

The isolate PpnIIT23 of *P. parasitica* race 0 used in our study was provided by the Tobacco Research Institute in Havana, Cuba. For inoculum preparation, sterilised V8 juice-impregnated toothpicks were placed onto potato dextrose agar plates and inoculated with a 5-mm plug from actively growing *P. parasitica* cultures. Plates were incubated for 14 days in the dark (27 °C) to ensure full oomycete’s colonisation.

Pot trials were conducted 21 d.p.ai. in a containment glasshouse (CIGB) at 25 °C. PVX-agroinfiltrated plants were 15 cm tall, with a stem diameter of 5 mm, and growing in plastic pots (20 cm diameter) filled with pasteurised soil. Each treatment (PVX.PxSDF2L1 or PVX.GFP) consisted of 12 plants (two plants per pot) and was replicated three times. Plants were hand-watered when required to maintain constant wet soil conditions.

Plants were inoculated by aseptically pushing *Phytophthora*-infested toothpicks into root systems near the base of the plant [40]. Uninfected toothpicks acted as controls. For measuring the stem lesions, a linear scale of 1–10 was chosen according to Csinos [27], where 1 refers to no disease or disease resistance, while 10 encompasses disease susceptibility and total mortality of the plants. The ratings were taken on stems at seven d.p.i.

### Data analysis

Data were analysed using the GraphPad Prism software version 8.0.2 for Windows. Analysis of variance (ANOVA) in conjunction with Tukey’s post-test at *P* < 0.05 as the significance level were performed to determine differences among treatments during the *Phytophthora* pot trials. Three replications of each treatment were performed, and similar results were obtained. The standard error of means was used to compare the replicates.

## Supplementary Information

**Additional file 1: Figure S1**. PVX-mediated recombinant GFP expression in *N. benthamiana*. Detection of green fluorescence in epidermal cells of systemically uninoculated leaves from PVX.GFP-agroinfected plants by CLSM, 21 d.p.ai. (+30). Systemically uninoculated leaves from plants challenged with PVX.PxSDF2L1 were used as the negative control. **Figure S2**. PCR to detect the PVX DNA vector. PCR products after 40 cycles with specific primers (5’-gaaacctcctcggattccat-3’; 5’-tctccaaatgaaatgaacttcc-3’) for a 312-bp fragment in the 35S promoter of cauliflower mosaic virus (p35S) in PVX-based binary vectors (PVX.PxSDF2L1 or PVX.GFP) on DNase I-digested total RNA (1 μg) isolated from the root of PVX.PxSDF2L1-agroinfected plants (lane 1-6); (+) *Agrobacterium tumefaciens* strain GV3101 cells carrying the PVX.PxSDF2L1 vector. M, 1 kb DNA ladder.

## Data Availability

The datasets used and/or analysed during the current study are available from the corresponding author on reasonable request.

## Abbreviations

SDF2: Stromal cell-derived factor 2
SDF2L1: Stromal cell-derived factor 2-like 1
ER: Endoplasmic reticulum
PVX: Potato Virus X
BiP: Hsp70 luminal binding protein
ER-QC: Endoplasmic reticulum quality control
ERAD: Endoplasmic reticulum-associated protein degradation
UPR: Unfolded protein response
PCD: Programmed cell death
PRR: Pattern recognition receptor
PTI: Pattern-triggered immunity
Xoo: *Xanthomonas oryzae* pv. *oryzae*
Bt: *Bacillus thuringiensis*
d.p.ai.: Days post-agroinfection
d.p.i.: Days post-inoculation
RACE: Rapid amplification of cDNA ends
LB: Luria Bertani
NaOAc: Sodium acetate
ANOVA: Analysis of variance
