# Correction to: Potato virus X-mediated constitutive expression of *Plutella xylostella PxSDF2L1* gene in *Nicotiana benthamiana* confers resistance to *Phytophthora parasitica* var. *nicotianae*

**DOI:** 10.1186/s12870-021-03341-7

**Published:** 2021-11-23

**Authors:** Ivis Moran-Bertot, Lianet Rodríguez-Cabrera, Orlando Borras-Hidalgo, Siliang Huang, Yunchao Kan, Denis J. Wright, Camilo Ayra-Pardo

**Affiliations:** 1 Plant Division, Centre for Genetic Engineering and Biotechnology (CIGB), 10600 Havana, Cuba; 2 Shandong Provincial Key Laboratory of Microbial Engineering, School of Biotechnology, Qi Lu University of Technology, Jinan, 250353 Shandong People’s Republic of China; 3 China-UK-NYNU-RRES Joint Laboratory of Insect Biology, Nanyang Normal University (NYNU), Nanyang, 473061 Henan People’s Republic of China; 4 Department of Life Sciences, Imperial College London, Silwood Park campus, Ascot, Berkshire, SL5 7PY UK

**Correction to: BMC Plant Biol 21, 78 (2021)**

**https://doi.org/10.1186/s12870-021-02854-5**

Following publication of the original article [1], an error was found in the Acknowledgement section. “Camilo Ayra-Pardo was a grantee of the Henan Science and Technology Department (Project no. HNGD2021049) in China.” Has to be added in the Acknowledgement section of the article.

The original article has been corrected.
