# Supplementary material for: Potato virus X-mediated constitutive expression of Plutella xylostella PxSDF2L1 gene in Nicotiana benthamiana confers resistance to Phytophthora parasitica var. nicotianae
Source: BMC Plant Biol. 2021 Feb 5;21:78. doi: 10.1186/s12870-021-02854-5 (PMC7866777; doi:10.1186/s12870-021-02854-5)
Supplement: Supplementary file 1 — Additional file 1: Figure S1. PVX-mediated recombinant GFP expression in N. benthamiana. Detection of green fluorescence in epidermal cells of systemically uninoculated leaves from PVX.GFP-agroinfected plants by CLSM, 21 d.p.ai. (+30). Systemically uninoculated leaves from plants challenged with PVX.PxSDF2L1 were used as the negative control. Figure S2. PCR to detect the PVX DNA vector. PCR products after 40 cycles with specific primers (5’-gaaacctcctcggattccat-3’; 5’-tctccaaatgaaatgaacttcc-3’) for a 312-bp fragment in the 35S promoter of cauliflower mosaic virus (p35S) in PVX-based binary vectors (PVX.PxSDF2L1 or PVX.GFP) on DNase I-digested total RNA (1 μg) isolated from the root of PVX.PxSDF2L1-agroinfected plants (lane 1-6); (+) Agrobacterium tumefaciens strain GV3101 cells carrying the PVX.PxSDF2L1 vector. M, 1 kb DNA ladder. [file 12870_2021_2854_MOESM1_ESM.docx]

Potato Virus X-mediated constitutive expression of *Plutella xylostella* PxSDF2L1 gene in *Nicotiana benthamiana* confers resistance to *Phytophthora parasitica* var. *nicotianae*

Ivis Moran-Bertot^1,♦^, Lianet Rodríguez-Cabrera^1,♦^, Orlando Borras-Hidalgo^1,♣^, Siliang Huang^2^, Yunchao Kan^2^, Denis J Wright^3,^* and Camilo Ayra-Pardo^2,^*

**Figure S1. PVX-mediated recombinant GFP expression in *N. benthamiana*.** Detection of green fluorescence in epidermal cells of systemically uninoculated leaves from PVX.GFP-agroinfected plants by CLSM, 21 d.p.ai. (+30). Systemically uninoculated leaves from plants challenged with PVX.PxSDF2L1 were used as the negative control.

**Figure S2.** **PCR to detect the PVX DNA vector.** PCR products after 40 cycles with specific primers (5’-gaaacctcctcggattccat-3’; 5’-tctccaaatgaaatgaacttcc-3’) for a 312-bp fragment in the 35S promoter of cauliflower mosaic virus (p35S) in PVX-based binary vectors (PVX.PxSDF2L1 or PVX.GFP) on DNase I-digested total RNA (1 μg) isolated from the root of PVX.PxSDF2L1-agroinfected plants (lane 1-6); (+) *Agrobacterium tumefaciens* strain GV3101 cells carrying the PVX.PxSDF2L1 vector. M, 1 kb DNA ladder.

**Figure S1**

**Figure S2**
